# Supplementary material for: Asymmetric structure of the native Rhodobacter sphaeroides dimeric LH1–RC complex
Source: Nat Commun. 2022 Apr 7;13:1904. doi: 10.1038/s41467-022-29453-8 (PMC8991256; doi:10.1038/s41467-022-29453-8)
Supplement: Supplementary file 1 — Supplementary Information [file 41467_2022_29453_MOESM1_ESM.pdf]

SUPPLEMENTARY INFORMATION

Asymmetric Structure of the Native *Rhodobacter sphaeroides* Dimeric LH1-RC Complex

K. Tani, R. Kanno et al.

Supplementary Table 1 Cryo-EM data collection, refinement and validation statistics.

	Native dimeric LH1-RC complex (EMDB-32192, PDB ID: 7VY2)	Monomeric LH1-RC ΔU complex (EMDB-32193, PDB ID: 7VY3)
**Data collection and processing**		
Magnification	96000	9600
Voltage (kV)	300	300
Electron exposure (e–/Å^2)	40	40
Defocus range (μm)	–1.0 to –2.9	–0.8 to –2.7
Pixel size (Å)	0.820	0.820
Symmetry imposed	C1	C1
Initial particle images (no.)	164496	337006
Final particle images (no.)	124916	124589
Map resolution (Å)	2.8	2.6
FSC threshold	0.143	0.143
Map resolution range (Å)	369–2.8	295–2.6
**Refinement**		
Initial model used (PDB code)	7F0L	7F0L
Model resolution (Å)	3.1	2.6
FSC threshold	0.5	0.5
Model resolution range (Å)	220–2.8	125–2.6
Map sharpening *B* factor (Å^2)	–61	–70
Model composition		
Non-hydrogen atoms	46314	18831
Protein residues	4534	1846
Ligands	201	86
B factors (Å^2)		
Protein	46.1	29.7
Ligand	52.7	31.0
R.m.s. deviations		
Bond lengths (Å)	0.005	0.006
Bond angles (°)	2.667	2.676
Validation		
MolProbity score	1.83	1.83
Clashscore	13.13	10.91
Poor rotamers (%)	1.62	2.30
Ramachandran plot		
Favored (%)	97.81	97.99
Allowed (%)	2.19	2.01
Disallowed (%)	0.00	0.00

Supplementary Table 2 Comparison of the distances of His–BChl(Mg) and BChl(Mg)–BChl(Mg) in LH1, LH2 and RC special pairs from various phototrophic bacteria.

LH1 or LH2	Distance of His(Nε2) to BChl–Mg (Å) α	Distance of His(Nε2) to BChl–Mg (Å) β	Distance of Mg–Mg (Å) Long	Distance of Mg–Mg (Å) Short	PDB ID
Rba. sphaeroides **(Dimer)**	**2.59**	**2.21**	**9.49**	**8.34**	**7VY2**
Rba. sphaeroides **(ΔU)**	**2.52**	**2.24**	**9.54**	**8.23**	**7VY3**
Rba. sphaeroides (Monomer)	2.58	2.20	9.55	8.37	7F0L
Rsp. rubrum (LH1)	2.27	2.03	9.34	8.51	7EQD
Rps. palustris (LH1-W)	2.93	2.71	9.61	8.2	6Z5S
Tch. tepidum (LH1)	2.19	2.19	8.88	8.72	5Y5S
Trv. strain 970 (LH1)	2.33	2.31	8.90	8.46	7C9R
Blc. viridis (LH1)	2.54	2.25	8.8	8.5	6ET5
Rfx. castenholzii (B880)	2.32	2.29	9.5	9.3	5YQ7
Rbl. acidophilus (B850)	2.34	2.34	9.5	8.8	1NKZ
Phs. molischianum (B850)	2.27	2.32	9.2	8.9	1LGH

RC (special pair)	L-subunit	M-subunit	BChl *a* (L)–BChl *a* (M)	PDB ID
Rba. sphaeroides **(Dimer:A)**	**2.24**	**2.15**	**7.80**	**7VY2**
Rba. sphaeroides **(Dimer:B)**	**2.20**	**2.15**	**7.77**	**7VY2**
Rba. sphaeroides **(ΔU)**	**2.24**	**2.37**	**7.76**	**7VY3**
Rba. sphaeroides (RC-only)	2.27	2.06	7.84	2J8C
Rsp. rubrum	2.09	2.12	7.76	7EQD
Rps. palustris	2.73	2.74	7.69	6Z5S
Tch. tepidum	2.17	2.19	7.87	5Y5S
Trv. strain 970	2.33	2.31	7.65	7C9R
Blc. viridis	2.36	2.35	7.83	6ET5

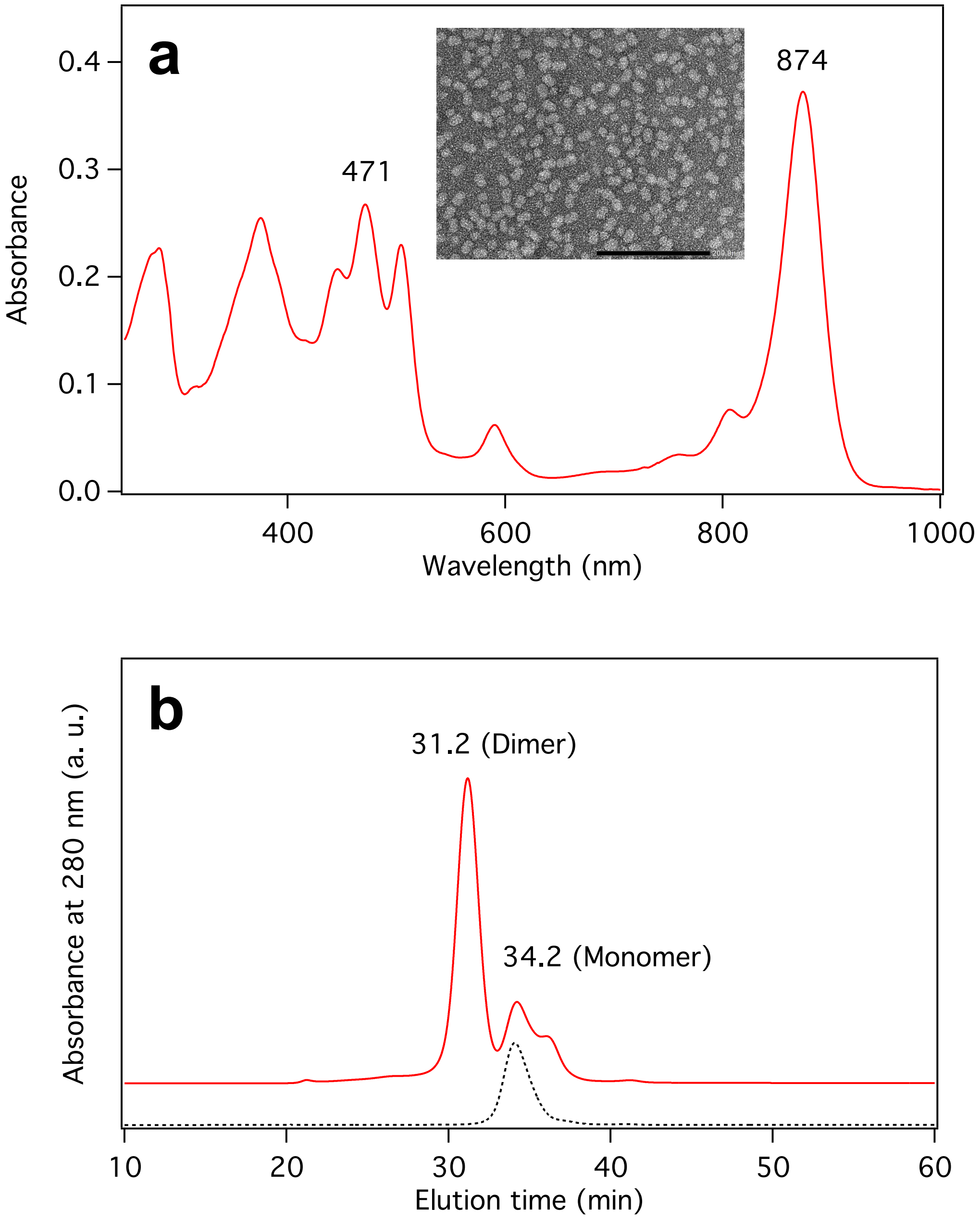

Supplementary Fig. 1 Absorption spectrum and gel-filtration chromatogram of the native *Rba. sphaeroides* IL106 dimeric LH1-RC complex. (a) Absorption spectrum of the purified dimeric LH1-RC at room temperature. Inset shows negatively stained LH1-RC particles obtained with 0.04 mg/mL LH1-RC in 20mM Tris-HCl (pH7.5) containing 0.05% DDM. Scale bar: 200 nm. **(b)** Gel-filtration chromatogram of the dimer-rich LH1-RC purified from DEAE chromatography using TSKgel G4000SW (7.5 mm I.D. × 60 cm, TOSOH) with 20 mM Tris-HCl buffer (pH 7.5) containing 200 mM NaCl and 0.05 % w/v DDM at flow rate of 1 mL/min at room temperature. Dashed black line shows the chromatogram of a highly purified monomeric LH1-RC.

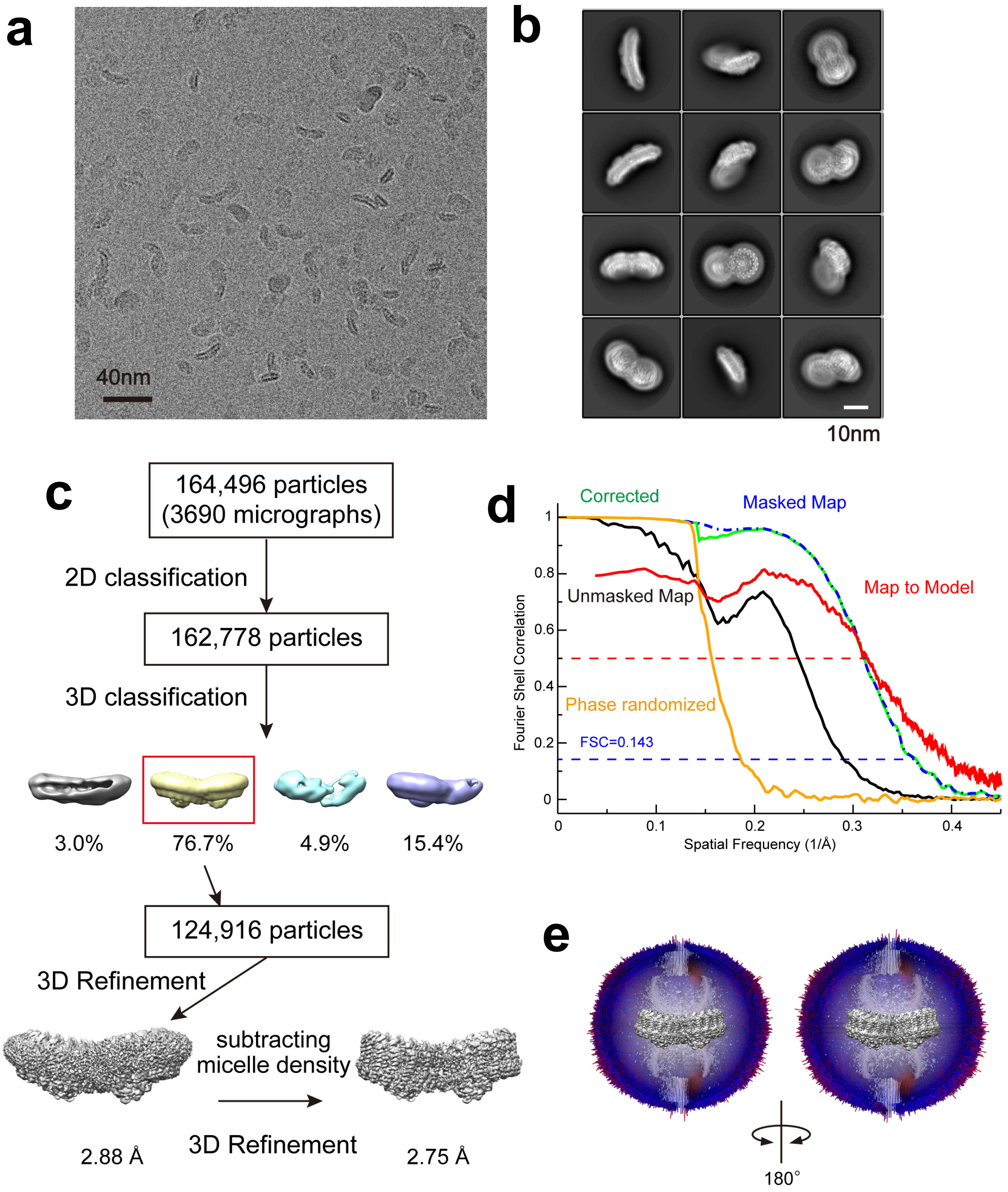

Supplementary Fig. 2 Structure determination of the native *Rba. sphaeroides* IL106 dimeric LH1-RC complex. **(a)** A representative cryo-EM micrograph. **(b)** Representative 2D class averages processed from the micrographs of LH1-RC. **(c)** Image processing flow of 3D classification and reconstruction. **(d)** The Fourier shell correlation (FSC) plots of the cryo-EM map (unmasked: black, masked: blue, phase randomized corrected: green, phase randomized: orange) and the FSC plot of the model versus the final map (red) are superimposed. **(e)** Angular distribution of reconstructed particles in the C1 map of dimeric LH1-RC complex. For clarity, the front half of angular distribution is removed.

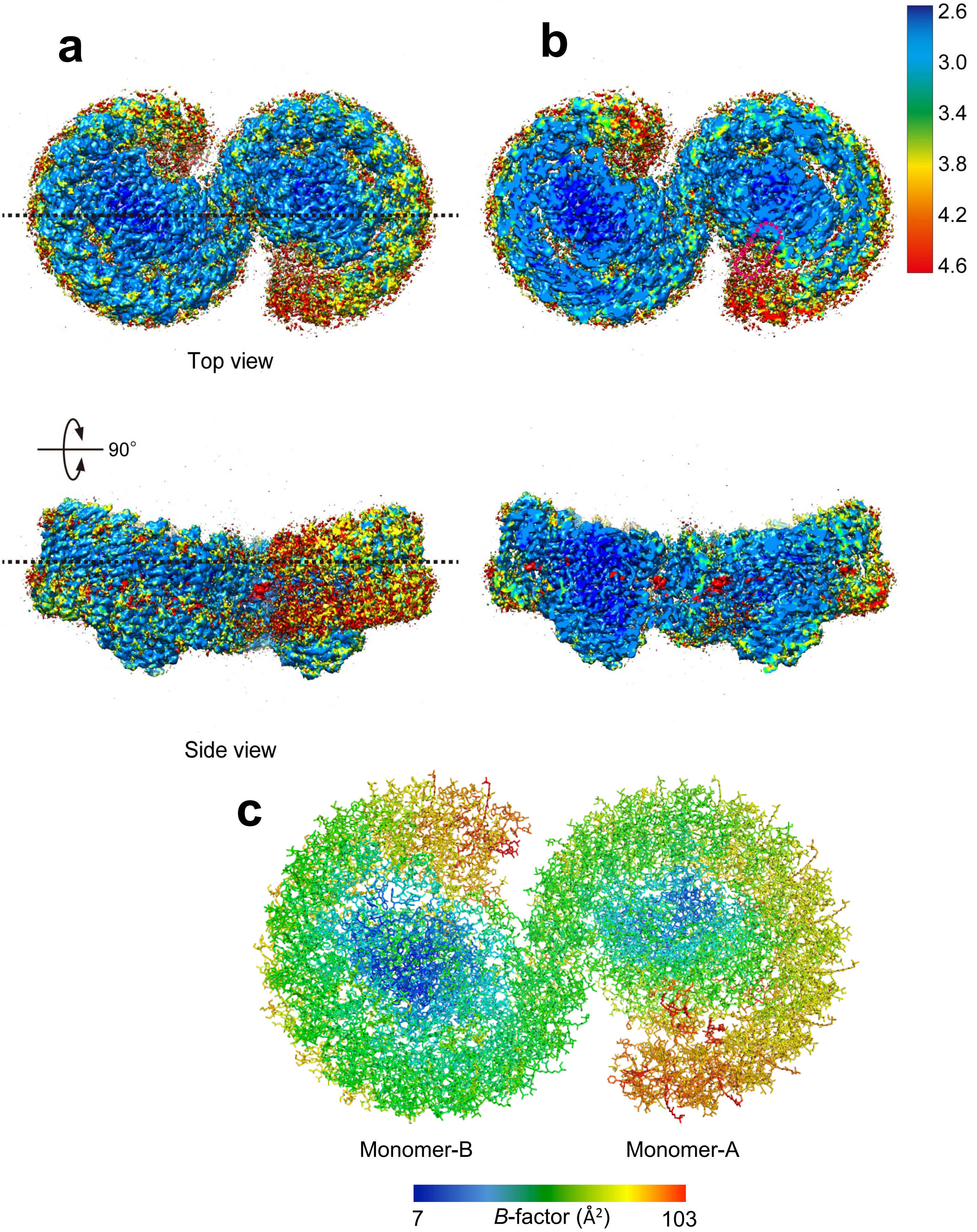

Supplementary Fig. 3 Local resolution and *B*-factor distributions of the native dimeric LH1-RC structure. **(a)** Top (periplasm) and side views of local resolution distributions. Dotted lines indicate cross sections. **(b)** Central crossing section views of local resolution distributions for the panels on the left-hand sides. The map color codes represent the resolutions from 4.6 Å (red) to 2.6 Å (blue) as indicated in the color bars. **(c)** Top view of *B*-factor distribution from periplasmic side. The color codes represent the *B*-values indicated in the color bar.

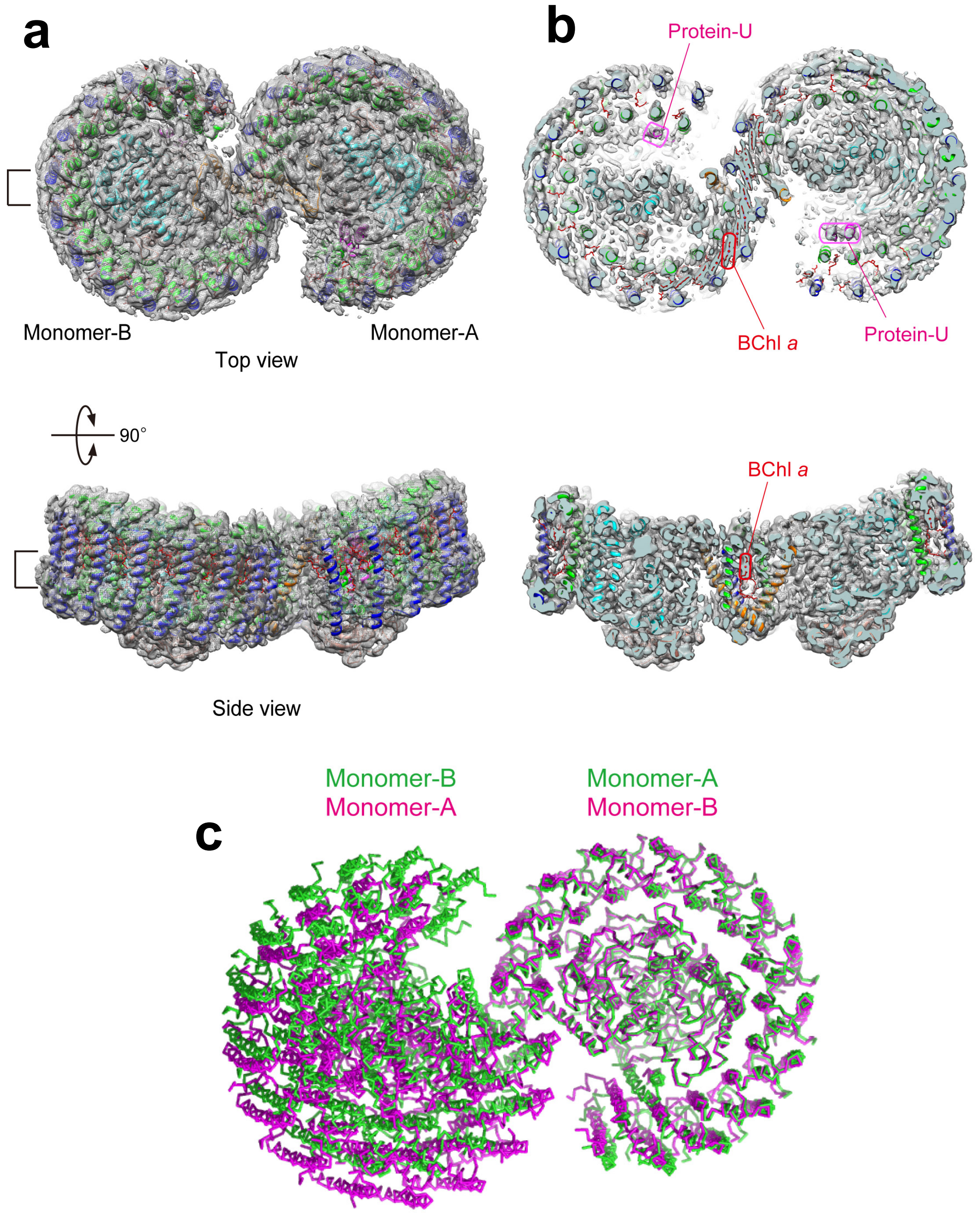

Supplementary Fig. 4 Overall structure of the native dimeric LH1-RC complex. Cartoon representation of the complex with cryo-EM unsharpened density map shown in gray mesh. **(a)** Top view from periplasmic side and side view parallel to the membrane plane. Each bracket indicates the slab region. **(b)** Top and side views of the slabs in (a) along and perpendicular to the membrane plane. The color codes corresponding to polypeptides and BChl *a* are the same as in Fig.1. Regions indicated by pink circles correspond to BChl *a*. **(c)** Overlap view of the two dimers by superposition of Cα carbons of the RC L-subunit in monomer-A (green) with those in monomer-B (180° rotated, magenta), showing asymmetric positions between the two monomers.

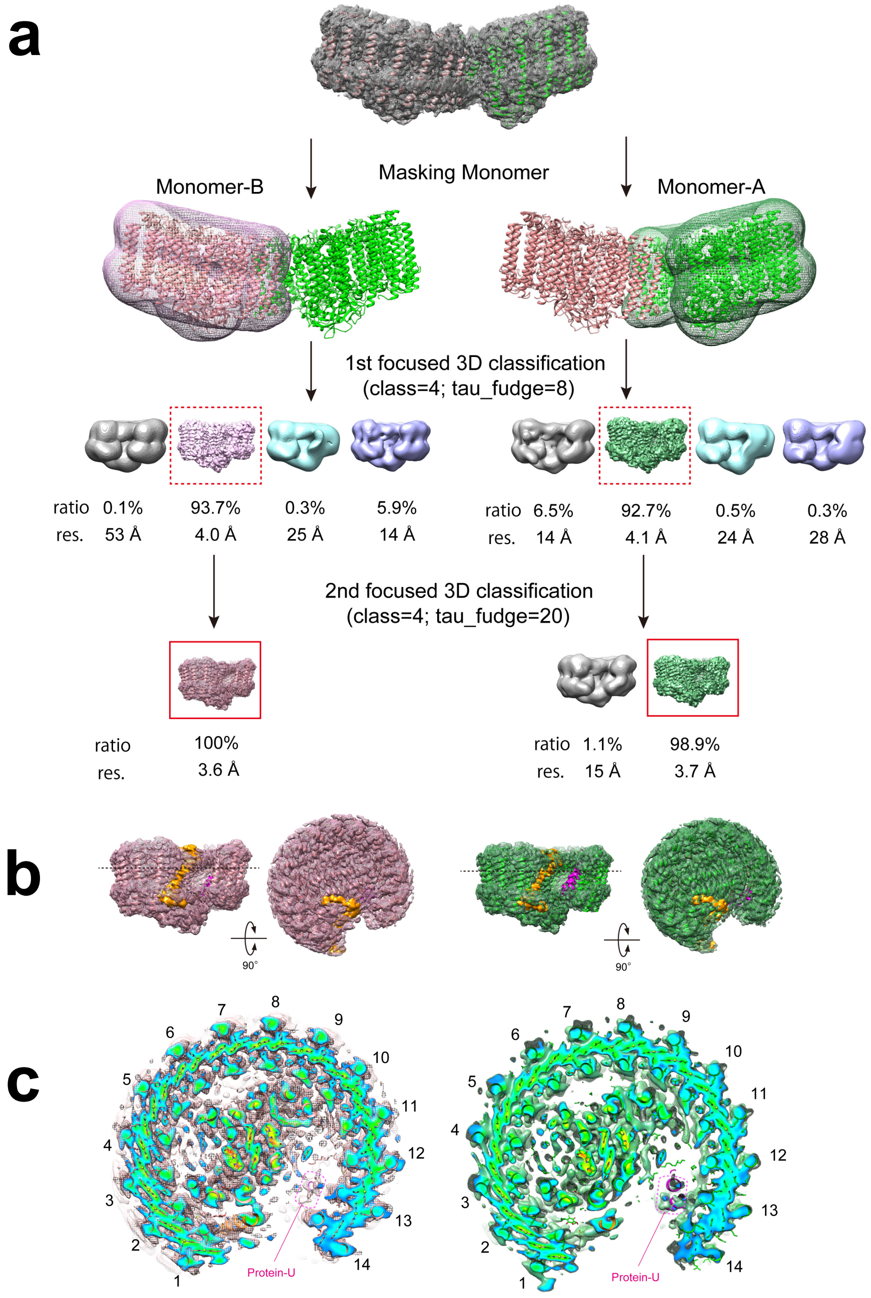

Supplementary Fig. 5 Focused 3D classification of the monomers in the native dimeric LH1-RC complex. **(a)** Focused classifications of the Monomer-A (green) and -B (pink) into 4 groups, respectively. The numbers below each class indicate resolution and ratio of the particles belonging to each class. **(b)** Top and side views of each major monomer map (red solid square in **(a)**) is superimposed on models of Monomer-A and -B, respectively. Each corresponding model (Monomer-A: green and Monomer-B: salmon) is covered with the corresponding map from individual reconstruction (Monomer-A: forest green mesh and Monomer-B: pink mesh). Magenta and orange surface represent the region of protein-U and PufX, respectively. For clarification, models of protein-U and PufX are hidden. **(c)** Top views of the slabs in **(b)** perpendicular to the membrane plane. Cross sectioned line is shown as a dotted line in **(b)**. The color codes of ribbon and mesh are the same as in **(b)**. Capped surface color corresponds to the map density value (from blue to red).

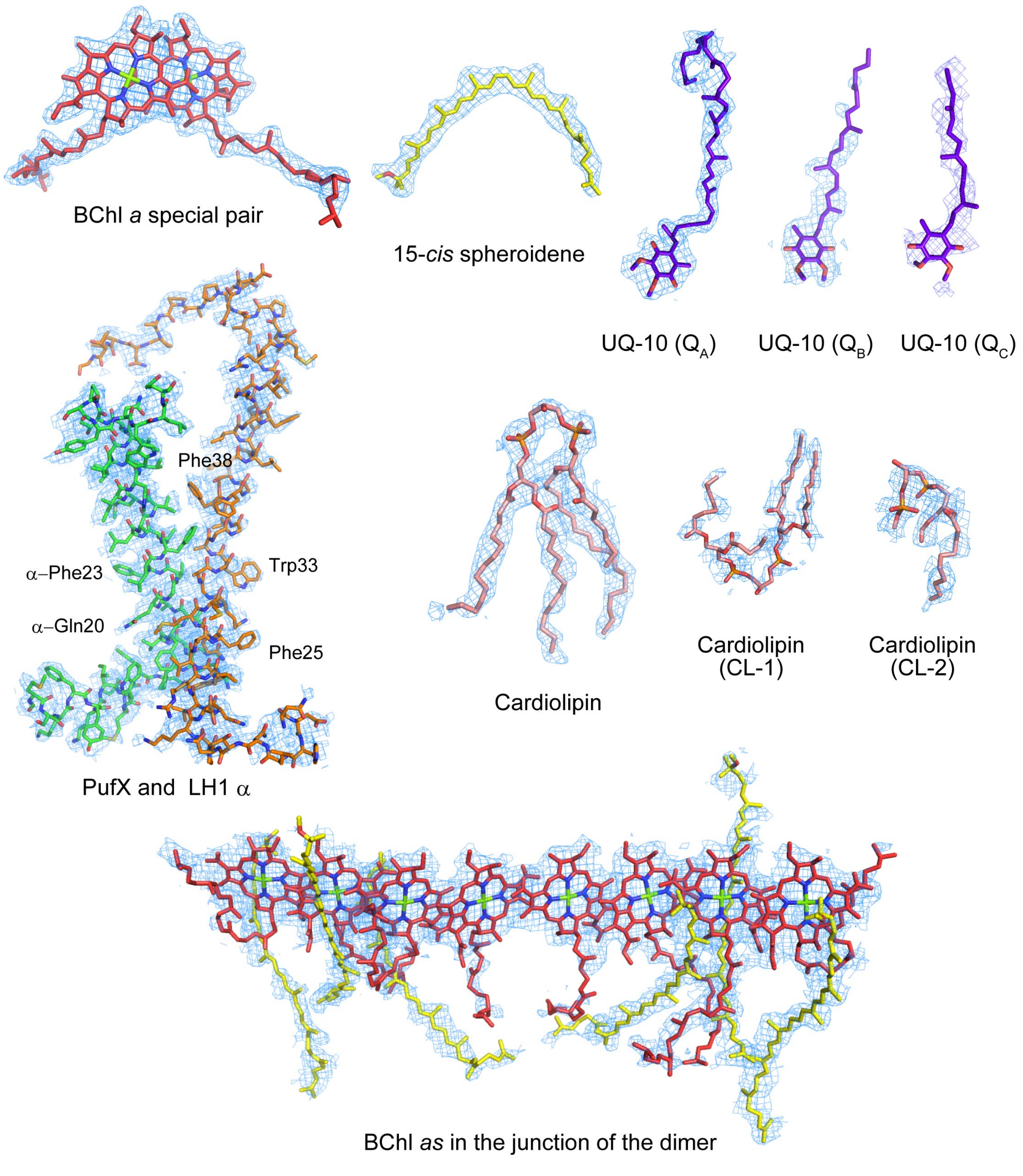

Supplementary Fig. 6 Cryo-EM densities and structural models in the dimer of the *Rba. sphaeroides* IL106 LH1-RC complex. The color codes of polypeptides are the same as in Fig. 1. The density maps are shown at a contour level of 4.0σ.

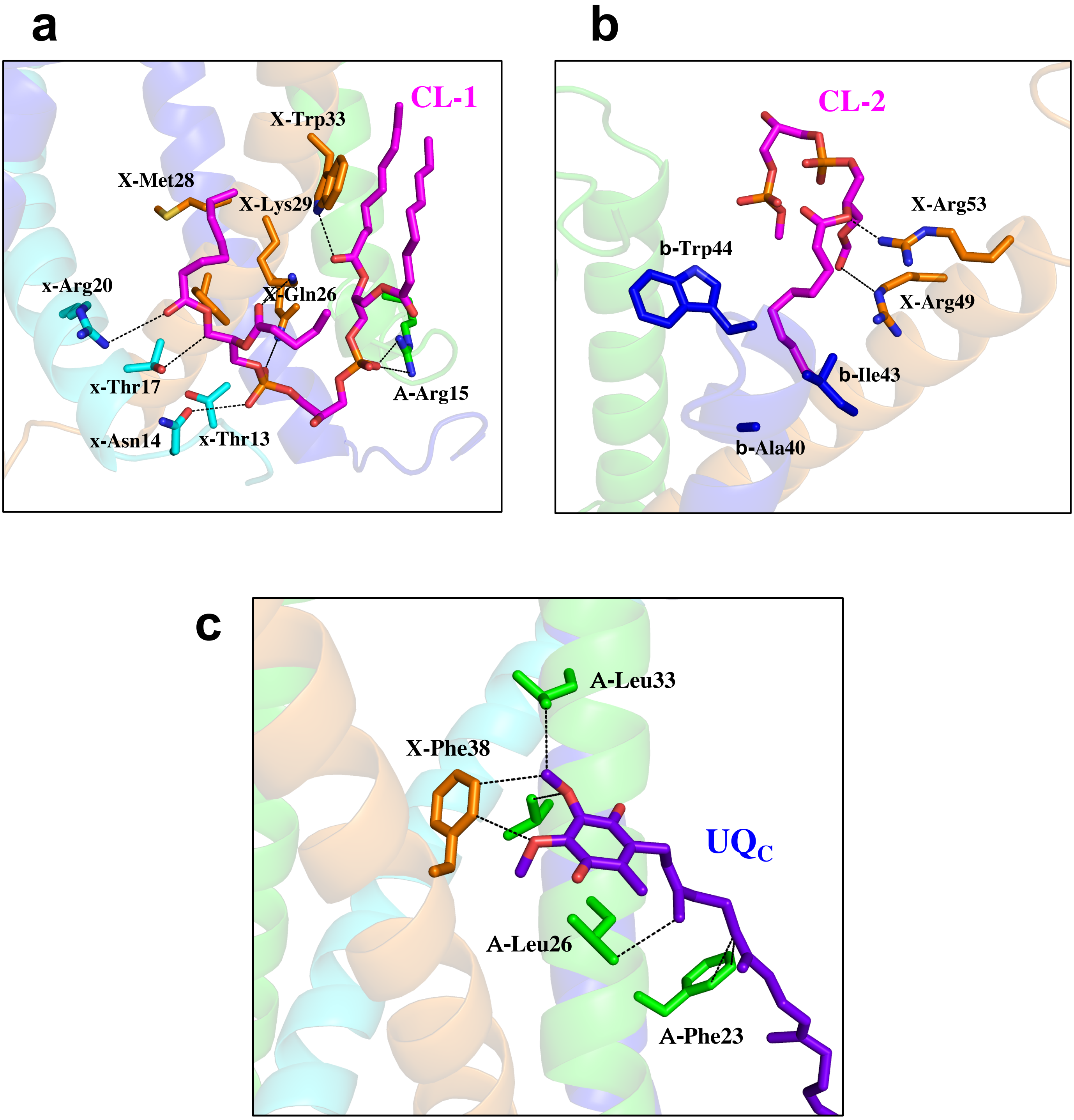

Supplementary Fig. 7 Interactions of the cardiolipin (CL) and UQ molecules in the dimer junction with nearby polypeptides. (a) Close contacts (<4.0 Å) between CL-1 (magenta sticks) and two PufX polypeptides (orange and cyan with chains ID: X and x) on the cytoplasmic side. Dashed lines indicate possible hydrogen bondings. **(b)** Close contacts (<4.0 Å) of CL-2 (magenta sticks) with a PufX (orange, chain ID: X) and a β-polypeptide (blue, chain ID: b) belonging to another monomer on the periplasmic side. **(c)** Close contacts (<4.0 Å, dashed lines) of UQ$_C$ (purple sticks) with a PufX (orange, chain ID: X) and an α-polypeptide (green, chain ID: A) in the transmembrane region.

a

```
              31  34
PufX:     QMMKGAGWAGGVFFGT
α-chain:  RRVFVAQGVFLFLLAV
             18  21
```

b

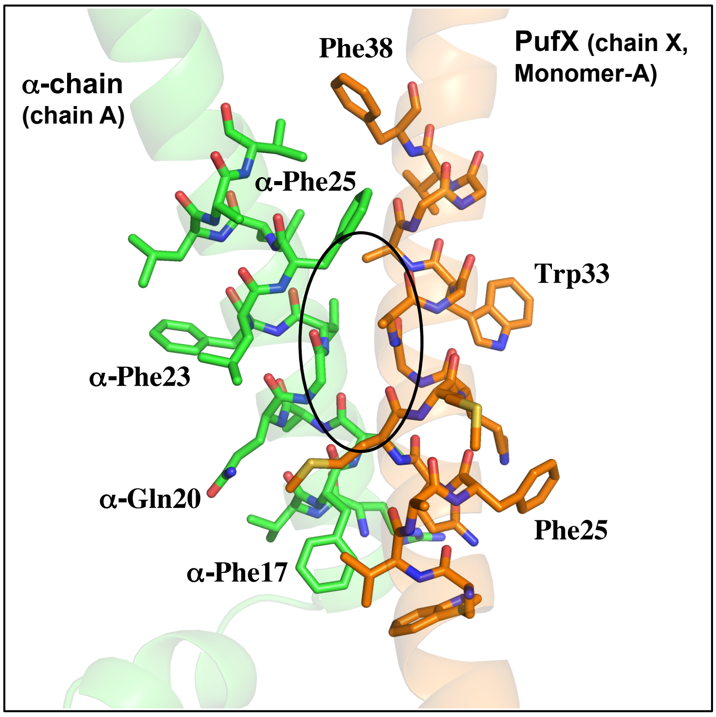

c

```
                           10        20        30        40        50        60        70        80
Rba. sphaeroides   MADKTIFNDHLNTNPKTNLRLWVAFQMMKGAGWAGGVFFGTLLLIGFFRVVGRMLPIDENPAPAPNITGALETGIELIKHLV
Rba. azotoformans  MADKTIFDDHLKTNPKTNLRLWVAFQMMKGAGWAGAVFFGTLLMIGFFRVLGRALPIDENPAPAPNLTGALETGIELIKHLV
Rba. veldkampii    MAEKHYLDGATKVG----MATMGAAAMGKGMGITAVVFFGTVFFVVALAFIGQFLPDRSREAPYPNTIFQVNDIDGTVDGKYTRFAN
Rba. capsulatus    MSMFDKPFDYENGS-KFEMGIWIGRQMAYGAFLGSIPFLLGLGLVLGSYGLGLMLPERAHQAPSPYTTEVVVQHATEVV
Rba. blasticus     MAEYNYSHEPNAVI---NLRVWALGQMVWGAFLAAVGVVVVICLLVGTYLAGLLLPEQSKQAPSPYGALEIVQTIDVA
                   *                         *  *                     *  **     ** *
```

d

```
                                 10        20        30        40        50
Rba. sphaeroides    -----MSKFYKIWMIFDPRRVFVAQGVFLFLLAVMIHLILLSTPSYNWLEISAAKYNRV
Rba. azotoformans   -----MAKFYKIWMIFDPRRVFVAQGVFLFLLAVMIHLILLSTPSYNWLEISAAKYNRVAVAE
Rba. veldkampii     -----MSKFYKIWLIFDPRRVFVAQGVFLFLLAVMIHLMLLSNPGFNWLDISGVKYERVAAE
Rba. capsulatus     -----MSKFYKIWLVFDPRRVFVAQGVFLFLLAVLIHLILLSTPAFNWLTVATAKHGYVAAAQ
Rba. blasticus      -----MSKFYKIWQVFDPRRVFVAQGVFLFLLAVMIHLILLSKPDYNWLDVGTAKYGRGEAAAVVTP
Tch. tepidum        MFTMNAN-LYKIWLILDPRRVLVSIVAFQIVLGLLIHMIVLST-DLNWLDDNIPVSYQALGKK
Trv. strain 970 A1  MNAKSFDGMHKLWMIMNPVSTLWAIFIFQIFLGLLIHMVVLSS-DLNWHDDQIPVGYQLQGETLPVNLEMKAAQ
Trv. strain 970 A3  MNAKSFDGMHKLWMIMNPVSTLWAIFIFQIFLGLLIHMVVLSS-DLNWHDDQIPVGYQLQGETLPVNLEMKAALKDAQ
Blc. viridis        MATEYRTASWKLWLILDPRRVLTALFVYLTVIALLIHFGLLSTDRLNWWEFQRGLPKAA
Rsp. rubrum         --------MWRIWQLFDPRQALVGLATFLFVLALLIHFILLSTERFNWLEGASTKPVQTSMVMPSSDLAV
                                *    *                 **   **    **
```

Supplementary Fig. 8 Interactions between PufX and α-polypeptide in the transmembrane region. (a) Sequence alignment of the crossing interface between the two polypeptides with a Gly- and Ala-rich stretch (red fonts) in PufX facing to a short segment (VAQGV, red fonts) in the α-polypeptide. **(b)** Contact interface (elliptical box) between the two polypeptide grooves formed by residues with the smallest side chains (essentially Gly) whereas most bulky side chains point outward the interface. **(c)** Sequence alignments of the PufX from five *Rhodobacter* species showing a conserved stretch rich in Gly and Ala (red fonts). **(d)** Sequence alignments of the α-polypeptides for typical purple bacteria showing a conserved VAQGV segment in the *Rhodobacter* species**.** Symbol (*) denotes identical residues.

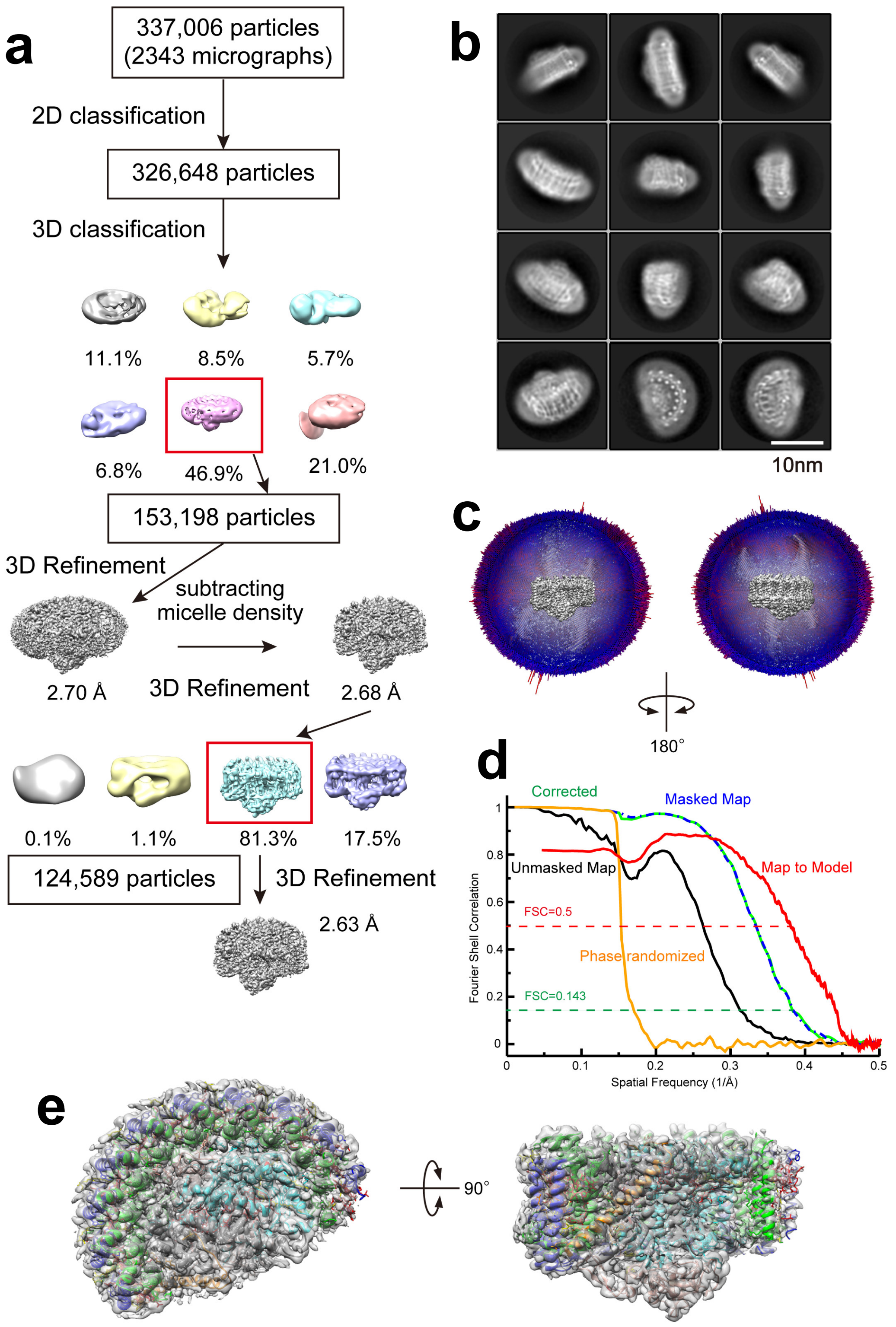

Supplementary Fig. 9 Structure determination of the monomeric LH1-RC ΔU complex. (a) Image processing flow of classification and 3D reconstruction. **(b)** Representative 2D class averages of ΔU LH1-RC monomer. **(c)** Angular distribution of reconstructed particles in the C1 map of ΔU LH1-RC complex. For clarity, the front half of angular distribution is removed. **(d)** FSC plots of the cryo-EM maps (unmasked: black, masked: blue, phase randomized corrected: green, phase randomized: orange) and the FSC plots of the model versus the final map (red) are superimposed. **(e)** Cartoon representation of the complex with density shown in gray mesh. Top view from periplasmic side and side view parallel to the membrane plane.

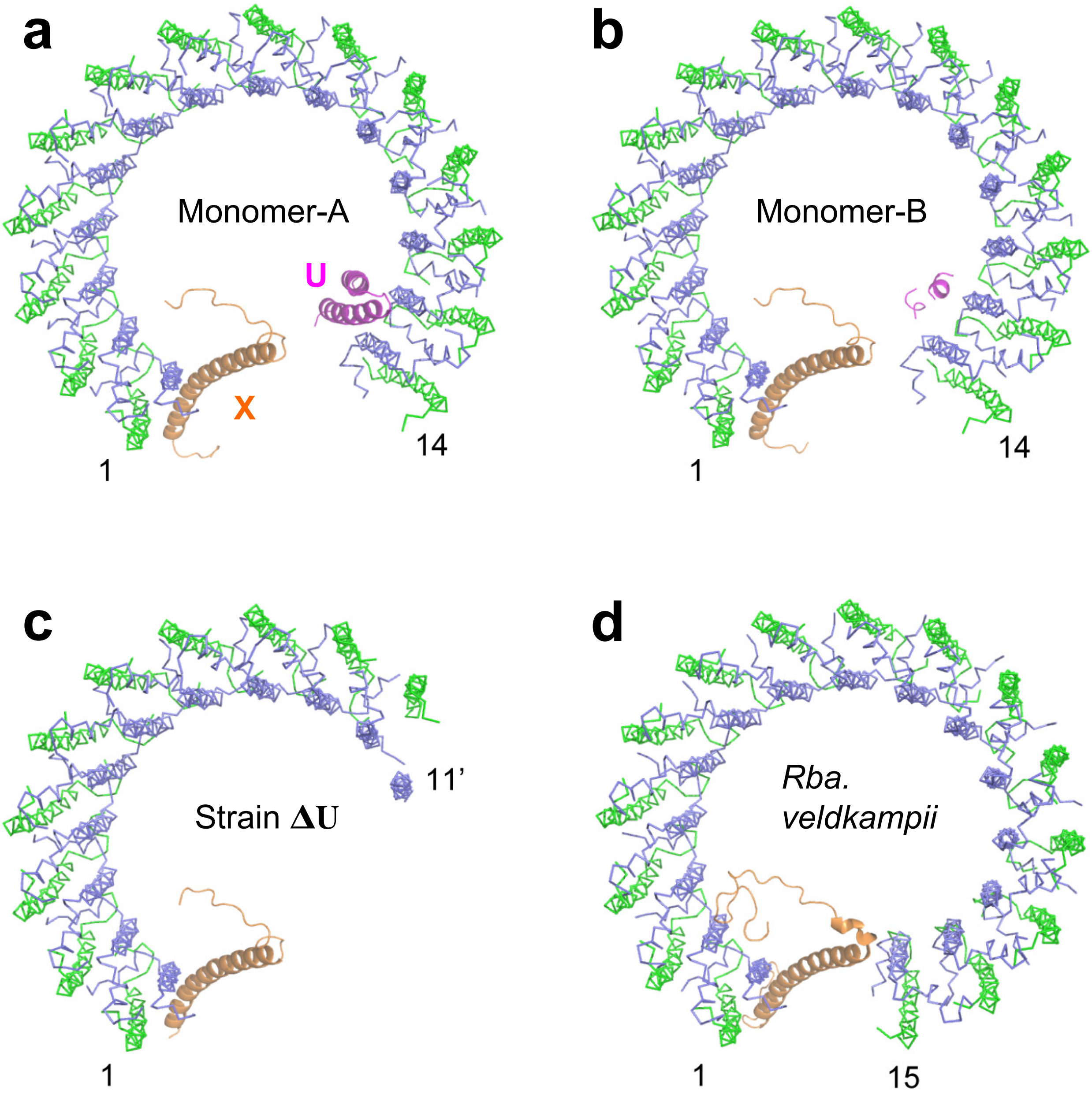

Supplementary Fig. 10 Comparison of the LH1 ring structure determined in this work with those reported in other LH1-RC-PufX structures. **(a)** Monomer-A of the dimer in this work (LH1-α, green ribbon; LH1-β, slate-blue ribbon; Puf-X, orange cartoon; Protein-U, magenta cartoon). **(b)** Monomer-B of the dimer in this work. **(c)** ΔU monomer lacking protein-U in this work. **(d)** Monomer in *Rba. veldkampii* (PDB 7DDQ). For clarification, RCs are not shown.

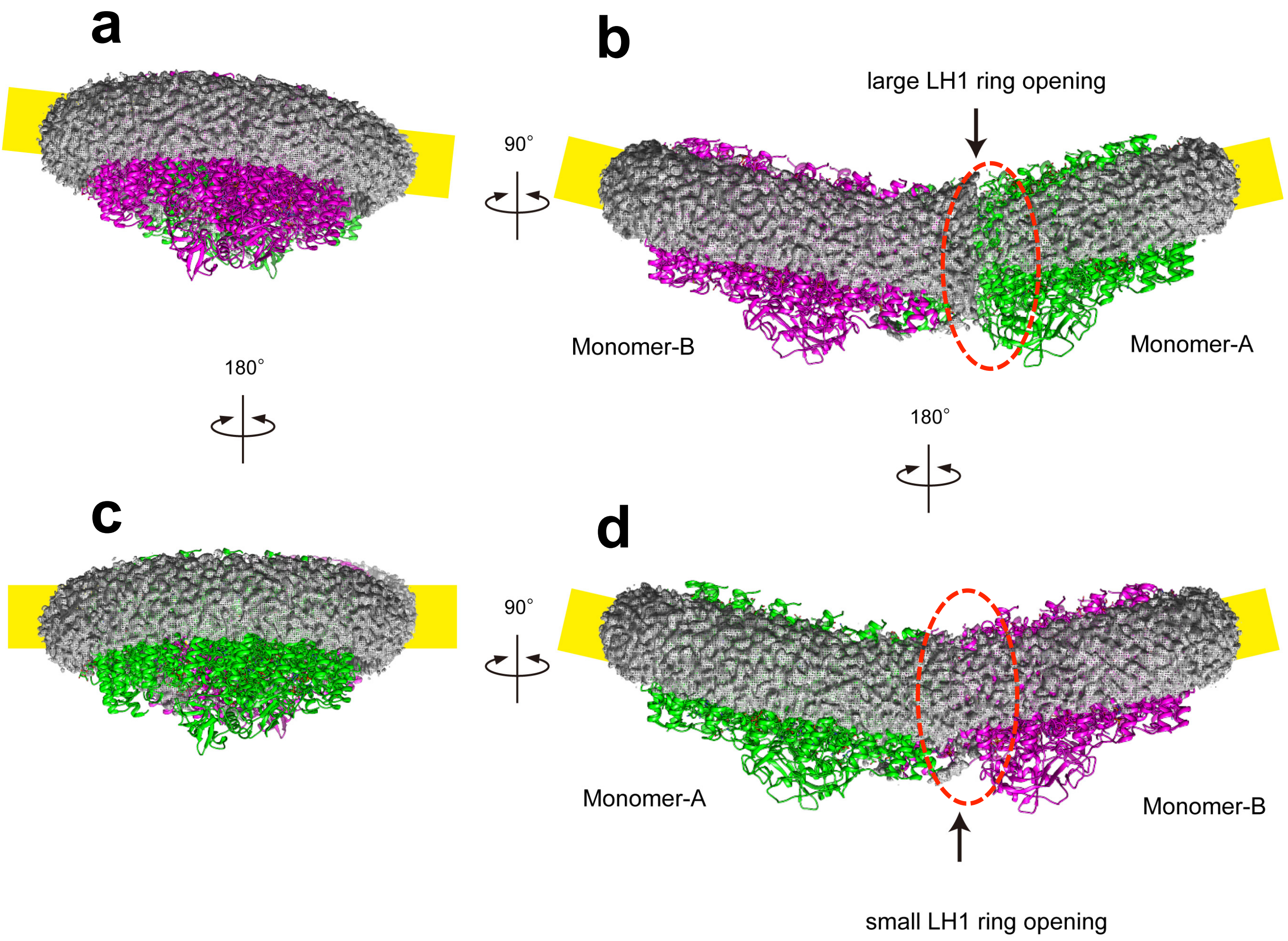

Supplementary Fig. 11 Asymmetric distributions of lipid/micelles around the LH1-RC dimer. Monomer-A, green cartoon; monomer-B, magenta cartoon; density maps for lipid/micelles, gray mesh; deduced membrane boundary, yellow box. **(a)** Side view from monomer-B along the long axis of dimer. The deduced membrane boundary is distorted. **(b)** Side view of the dimer with monomer-A on the right-hand side showing a larger LH1-ring opening (red dashed ellipse). The lipid/micelle belt is disconnected and the deduced membrane boundary is kinked by an angle of ~158º. **(c)** Side view from monomer-A along the long axis of dimer. The deduced membrane boundary is nearly flat. **(d)** Side view of the dimer with monomer-B on the right-hand side showing a smaller LH1-ring opening (red dashed ellipse). The lipid/micelle belt is continued and the deduced membrane boundary is kinked by an angle of ~152º.
